# CAHRD Consultation 2014: the 10-20 year Horizon Introduction and Overview – as circulated to Consultation participants

**DOI:** 10.1186/1753-6561-9-S10-S2

**Published:** 2015-12-18

**Authors:** SB Squire

**Affiliations:** 1 Centre for Applied Health Research & Delivery, Liverpool School of Tropical Medicine, Pembroke Place, Liverpool L3 5QA

## Abstract

The overall aim of the 2014 Consultation is to bring together internal and external partners to help shape the strategic direction for CAHRD over the 10 to 20 year horizon. Our strategic thinking will be guided by our vision of a healthy future for low and middle income populations and our mission to transform health systems to improve the health of these populations. Partnership between northern and southern institutions is integral to this work and critical in the consultation process. The Consultation considers four selected areas of the current work of CAHRD: Lung Health, Maternal & Newborn Health, Neglected Tropical Diseases, and Health Systems. We aim to foster dialogue and learning between these and across contexts and disciplines. The major challenges that will need to be addressed over the next 10 to 20 years will be scoped and pathways to possible solutions proposed. The overall vision is a process of co-production of knowledge

## Aim and objectives of the 2014 CAHRD Consultation

The overarching **Aim** of the 2014 CAHRD Consultation is to scope likely challenges on the 10 to 20 year horizon for a selected range of health topics: Lung Health (LH), Maternal & Newborn Health (MNH), Neglected Tropical Disease (NTD), and Health Systems (HS) (see **Workstreams** below), and to plan pathways towards solutions for these challenges. This time scale was chosen in recognition of the fact that research aimed at providing new knowledge to overcome challenges and bottle-necks in health improvement takes time to frame, conduct, analyse, synthesise and use effectively in policy and practice.

The specific **Objectives** of the 2014 Consultation are to:

i. present concrete options for the further growth and development of work within the selected Workstreams

ii. identify cross-cutting themes and areas for collaboration across and between the Workstreams

iii. identify areas for collaboration between the 2014 Workstreams and other on-going Applied Health Research & Delivery Activities

iv. strengthen existing collaborations and forge new collaborations with global partners

The **Workstreams** of the 2014 Consultation were selected from the full portfolio of Applied Health Research & Delivery co-ordinated by LSTM as examples of activity which:

i. represent a diverse range of health topics

ii. are from different places on the CAHRD spectrum of work (see Figs 3,4,5,6)

iii. engage a diverse range of global partners

In addition, some of the selected Workstreams are developing in collaboration with the methodological and disciplinary expertise of the University of Warwick (for examples: Mathematics & Epidemiology, Health Economics, Management Sciences).

As so many of the Applied Health Research & Delivery activities co-ordinated by LSTM fulfil all the criteria above, the final selection of the 2014 Workstreams was relatively arbitrary, with some bias towards areas which have more recently become established. For this reason, a brief overview of example areas of activity which were not selected this year is included in this paper (see Section 6 below)

## Preparing for the 2014 CAHRD Consultation Meeting: aiming for co-production of knowledge

Each Workstream was tasked with writing three discussion papers, each scoping major challenges that will need solutions over a 10 to 20 year time frame. Each paper:

• is based on an area of work to which LSTM and global partners have already made a contribution.

• includes a summary of the evidence to date – where possible and appropriate includes reference to available systematic reviews.

• is linked to an emerging international policy agenda or strategy.

• outlines a strategic approach that the Collaboration is well-placed to take forward.

Between February and May 2014, drafts of the 12 discussion papers have been shared for comment and editing across all Workstreams. This has provided an internal and external review process and has promoted dialogue across and between the Workstreams about disciplinary approaches, contexts and health areas. It has also permitted us to begin the identification of some emerging cross-cutting themes and priorities (see Section 7 below).

**Figure 1:**
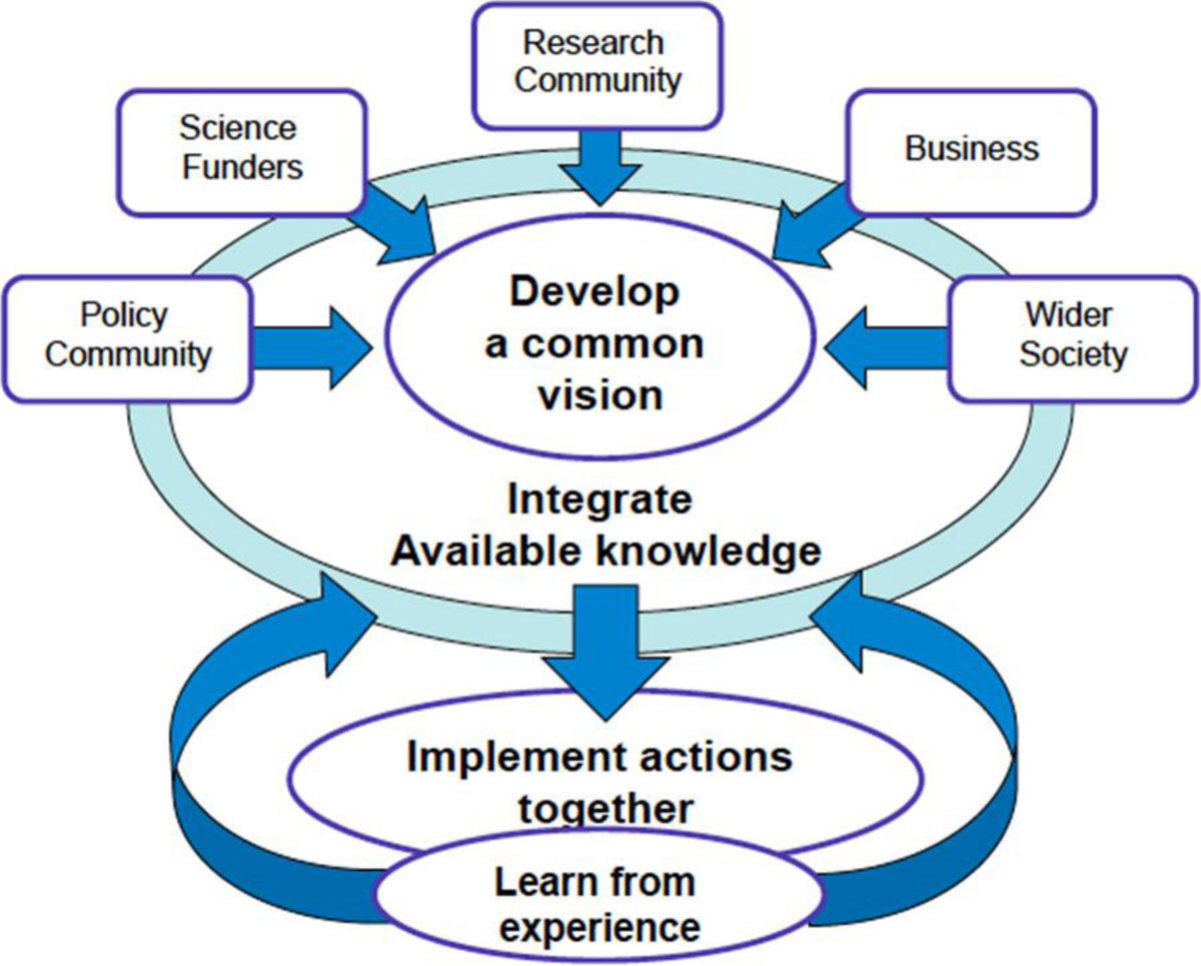
Co-production of knowledge (source: FutureEarth Research for Global Sustainability http://www.futureearth.info/who-we-are)

The papers have been circulated to the Consultation participants as part of the ongoing process of facilitating discussion within the meeting. They are not standalone, finalised outputs in their own right, but rather a means of eliciting perspectives from a relevant spectrum of stakeholders as illustrated in Figure 1 and recently advocated by Sheikh et al.[1] They are a starting point for discussion.

## The process of the 2014 CAHRD Consultation Meeting: sequence of discussion and flow of ideas and information

**Figure 2:**
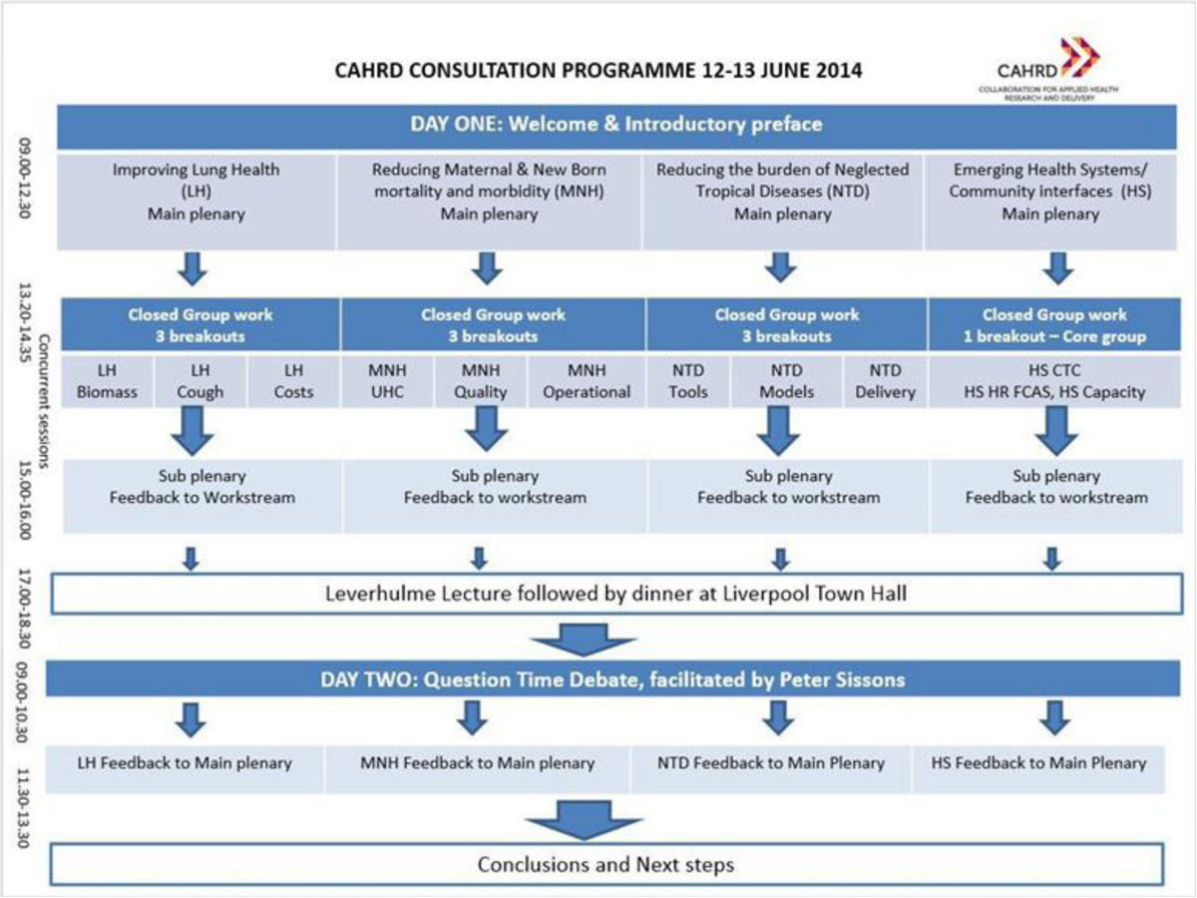
Overview of process for CAHRD Consultation programme

The overall sequencing of sessions and discussion is illustrated in Figure 2. The first day starts with brief presentations of the discussion papers in plenary with some initial commentary from selected members of the invited external panel. This is followed by in-depth discussion in groups which are reported back through sub-plenaries. By the end of the first day each Workstream will have a draft summary of progress and next steps ready for presentation back to the final plenary session at the end of the following morning. Between those two steps there is the opportunity to reflect and refine ideas in the light of the content of the Leverhulme Lecture in the evening of day one and a Question Time debate which opens day two.

The Question Time debate gives the opportunity for people who have not been able to attend the Consultation in person to contribute to the debate by having their questions debated in public by an external panel. Questions have been solicited in advance of the meeting and will be posed to the Question Time panel. The panel has been selected to be a mixture of senior, experienced colleagues debating alongside younger investigators. Not all panellists will be expected to respond to all questions, and supplementary questions can be asked from the floor. The intention is to provide an opportunity for lively and open discussion to shed additional perspectives on the topics of all four Workstreams.

## Applied Health Research & Delivery: Definitions

**Figure 3:**
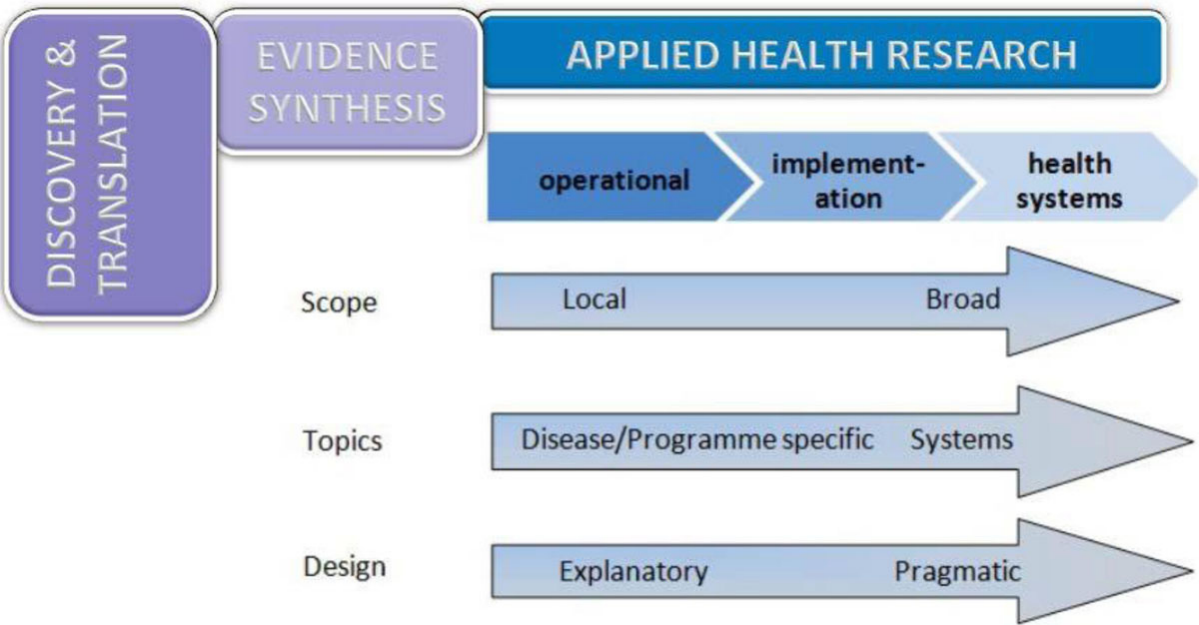
Defining Applied Health Research

**Figure 4:**
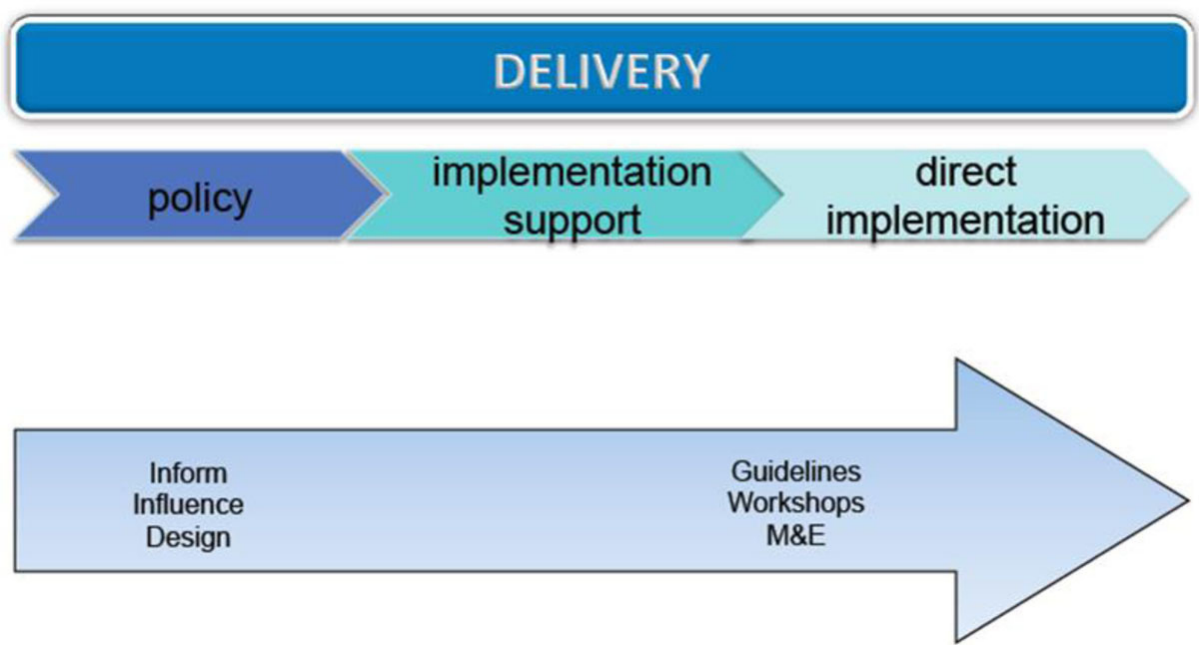
Defining Delivery

The domains of CAHRD activity include **Applied Health Research** and **Delivery**, which we broadly define as follows:

a) **Applied Health Research**. This encompasses the activities that help develop practical solutions to health needs and rights. It incorporates the spectrum of research that delivers policy-relevant evidence and includes operational, implementation, and health systems research. It is part of the value chain taking knowledge from Discovery and Translation and moving it into policy and practice as illustrated below and articulated in the recent literature.[2] These research domains are required to develop and synthesise a robust body of evidence to help drive policy and practice for health amongst poor and vulnerable populations in the developing world. It requires expertise from a range of disciplines to combine and integrate scientific, technological, economic and social insights. **Applied Health Research** includes a strong focus on taking interventions from regulatory approval through to field implementation (See Figure 3)

b) **Delivery**. This encompasses all the activities that develop individual and collective capabilities to implement best practices in health service delivery and multi-sectoral collaboration across a range of health scenarios. It will, *inter alia*, use evidence generated through **Applied Health Research** to support public health policy makers and providers in promoting health practices and addressing the delivery needs of poor populations, gender discrimination and health inequities. **Delivery** is primarily about supporting wide-scale implementation of interventions that have strong evidence of effectiveness and field-readiness (see Figure 4). It includes technical assistance in the form of guidance to meet the specific needs of (e.g.) a Ministry of Health through collaborative communication between a specialist team and the client.

**Figure 5:**
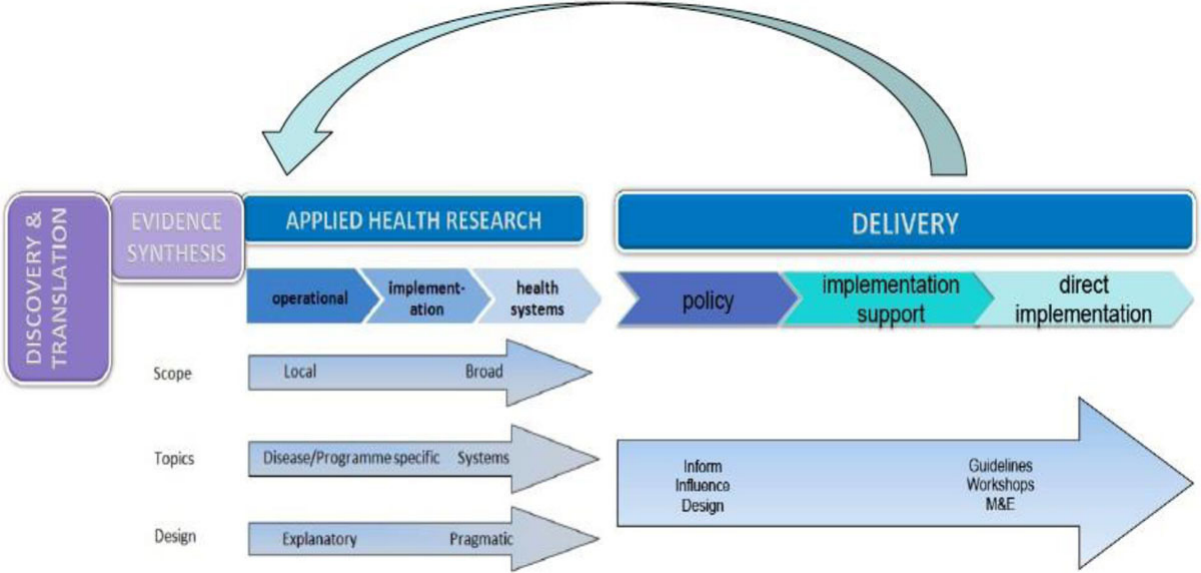
Applied Health Research and Delivery informing each other

Insights gained through health **Delivery** work clearly inform further **Applied Health Research** (See Figure 5):

The bi-directional flow of knowledge in relation to Applied Health Research has been represented in a linear fashion (see above). It is recognised, however, that in practice, multiple feedback loops operate in the many cycles of generating innovation, evidence and implementation. It is important to recognise that CAHRD fosters these feedback loops and maintains close and productive linkages with other domains of work, including Discovery and Translational Research.

## The four Workstreams (Lung Health, Maternal & Newborn Health, Neglected Tropical Diseases, & Health Systems) selected for the 2014 CAHRD Consultation

**Figure 6:**
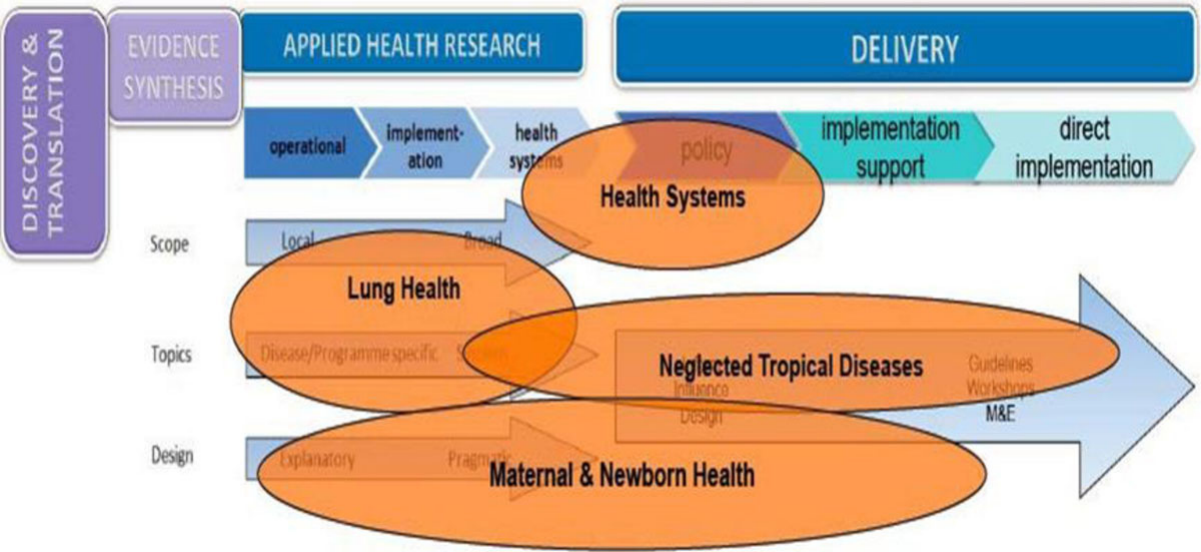
The four Workstreams (Lung Health, Maternal & Newborn Health, Neglected Tropical Diseases, & Health Systems) in relation to the spectrum of Applied Health Research & Delivery.

The four Workstreams were selected to represent a diversity of health topics where linkages into broad health systems work are being developed or are ongoing. The vision is that by focussing on these areas as examples, learning points for other areas could also be elicited. Inevitably a number of key areas of on-going work (see below) could not be covered in this first Consultation. There will be opportunities to focus on these in future Consultations. The precise placement of the Workstreams on the spectrum described above is difficult, as many encompass a considerable breadth of activity. Illustrative placements are depicted according to their main “centre of gravity” in Figure 6. Brief descriptions of each Workstream and some reasons for their selection then follow. Surnames of Workstreams leaders are included.

**a) Lung Health is an area where experience in chronic infectious disease control (mainly TB & HIV) provides a starting point for approaching chronic non-communicable disease control** (Mortimer, et al)

Consensus is building around the need for a greater focus on combating Non-Communicable Diseases (NCDs) such as diabetes, heart disease, chronic obstructive pulmonary disease (COPD) and asthma. These chronic illnesses require life-long clinical and public health management and are projected to contribute an increasing proportion of the global burden of disease in the next 10 to 20 years. LSTM and partners have attracted a number of staff interested in promoting improved prevention and management of chronic airways disease (COPD and asthma) in developing countries. With chronic cough as the commonest presenting symptom of TB, and a common feature of COPD (chronic bronchitis phenotype), asthma and bronchiectasis, there is an increasing need for health services to provide diagnostic and clinical management plans for these patients. These need to work in synergy with multi-sectoral development and evaluation of preventive strategies such as reduction of indoor air pollution and tobacco control.

**b) Maternal and Newborn Health remains in the spotlight** (Van den Broek et al)

The need to reduce maternal and child mortality and morbidity has been the subject of extensive high level discussion. In part this is driven by clarity that the reductions in mother and child mortality demanded by the Millennium Development Goals (MDGs), even though on track, will not be met by most countries by 2015. Major global health funders have signalled that maternal and child health programmes will remain among their highest priorities. The latest maternal global mortality figures were released in May 2014 and illustrate the need for accelerated action. Within child health, the main focus will be on the newborn with new targets and actions agreed to reduce neonatal deaths and stillbirths.

LSTM's Centre for Maternal & Newborn Health manages a large portfolio of work focussed on design, implementation and evaluation of interventions to improve availability and quality of care for mothers and babies in low and middle income countries.

**c) Neglected Tropical Diseases are nearing a period of transition in approaches needed for control** (Torr et al)

Huge gains have been made in the control of NTD's through the use of preventive chemotherapy in mass drug administration (MDA) programmes. These programmes have largely been delivered with a “vertical” approach and with specific diseases targets such as schistosomiasis, lymphatic filariasis and onchocerciasis. With the success of these programmes, some countries now face a future in which disease prevalence will fall and control will rely increasingly on interventions delivered through a more integrated approach within routine health services. Some countries, however, have only just started MDA programmes. These tend to be fragile or conflict affected states and success may not come so easily in these more challenging contexts. In addition there is an increasing recognition that some of the NTDs (e.g. trypanosomiasis) are not amenable to mass drug administration approaches, and will require multi-sectoral engagement including health, agriculture and veterinary services. LSTM has supported the science behind mass drug administration for NTDs over many years. There has also been substantial work at LSTM developing new diagnostics, drugs and insecticides for the control of NTD's. Many of these will be transitioning into field use in the next 10 to 20 years. These need to be evaluated in large scale population based studies in relevant disease endemic country settings. Evaluations will require health economic, logistics, delivery and social science inputs.

**d) Strengthened Health System approaches will be critical for delivery of health improvements in all disease-specific areas** (Theobald, MacPherson, Raven, Tolhurst et al)

Formal health systems serving poor populations in the developing world tend to be weak in components of governance, human resources, financing, essential technologies, and health information systems. Human resources are absolutely critical for improvements in health. With severe shortages of formally trained and skilled staff in all of the disease-specific areas described above, there has been a growing experience with the use of close-to-community providers or community health workers. Human resource constraints are particularly limiting in fragile, post-conflict states, where disease burdens are also high. In order to overcome these and other health system constraints, effective approaches to both individual and systems capacity strengthening are required.

## Examples of on-going activities co-ordinated by LSTM but not the focus of the 2014 Consultation

**Figure 7:**
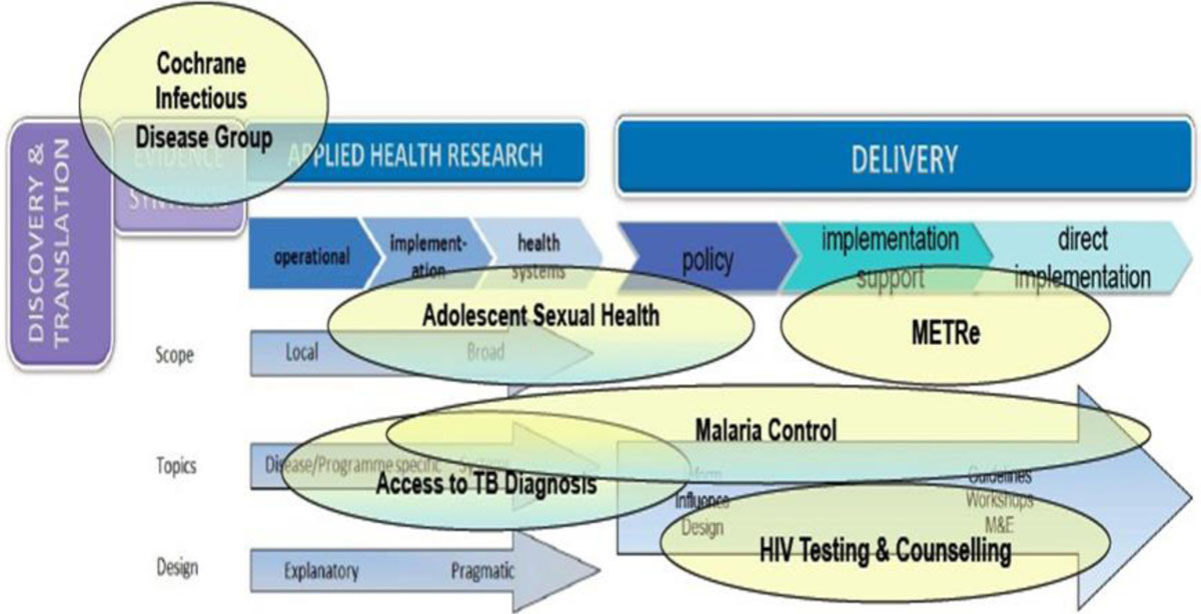
Examples of on-going activities co-ordinated by LSTM across the spectrum and in relation to Discovery, Translation, Evidence Synthesis

Inevitably, a number of key areas of on-going work could not be covered in the 1.5 days of this first CAHRD Consultation. However, one objective of the Consultation is to identify potential synergies between these activities and the Workstreams, and the intention is to return to these in future Consultations. As in Section 5 above, the precise placement of existing research groups and consortia on the spectrum of Applied Health Research & Delivery is difficult. Illustrative placements are depicted according to their main “centre of gravity” in Figure 7. Brief descriptions of selected areas of work then follow along with surnames of key investigators.

**a) Evidence Synthesis** (Garner et al)

Systematic reviews are central to translating research into policy and practice. LSTM has hosted the Cochrane Infectious Disease Group since 1996. The group consists of 17 editors and has published 144 reviews. It has made several contributions to policy development. Selected examples include a global change in the formula for oral rehydration salt;[3] the move from monotherapy to artesunate combination chemotherapy for malaria;[4] debate about the benefits of deworming for child health;[5] and the decentralisation of HIV treatment from hospitals to health centres.[6]

**b) METRe - development of innovative tools for monitoring of health programmes** (Valadez et al)

The multi-disciplinary METRe Group was created to provide technical assistance and build capacity in low resource countries on how to effectively collect, analyse and use quality data to guide decision making for health programmes and policies. The group's main scope of work includes the development of innovative tools for monitoring of health programmes, such as using Lot Quality Assurance Sampling (LQAS) to provide real-time district level health information at both the household and health facility level.[7-9]

The group specialises in the development and use of practical tools for assessment.[10] METRe is committed to developing local capacity to undertake this work, and uses a Learning By Doing approach for this purpose.[11] Research is an on-going component of the work of the METRe group which strives to ensure that monitoring strategies are evidence-based. Recent projects include:

• An evaluation of the effect of a results-based financing (RBF) intervention in Acholi region, Northern Uganda for DfID.

• Support to the government of South Sudan to strengthen systems for collecting health data using a mixture of Health Facility Surveys (HFS) community-based Household Surveys (HHS) and strengthening of HMIS data collection procedures.

• Support to the National Vector Borne Disease Control Program by working in 21 districts in Odisha State, India.

**c) Malaria** (Hemingway, Ranson, ter Kuile, et al)

The malaria research and delivery activities of LSTM have been aimed at improving control in high risk populations such as infants, young children, HIV-infected people. Both vector control and treatment approaches have been employed.

Malaria in pregnancy (MiP) can have devastating consequences in both mother and fetus. The MiP Consortium was initiated with funding from the Bill & Melinda Gates Foundation in 2008 and coordinated by a secretariat based at LSTM. It has provided key contributions to WHO's new global burden of MiP estimates [12]) and helped WHO and countries achieve a better understanding of the coverage and reasons behind the low uptake of existing interventions by pregnant women of existing interventions.[13-15] Through this work it provided the evidence that led WHO in 2013 to update its policy recommendation for the prevention of MiP, benefiting 39 countries in sub-Saharan Africa.[16] In June 2015 at WHO's Evidence Review Group on MiP the Consortium will present results of 4 multi-country prevention trials potentially leading to further policy change.

More recently the emphasis has shifted from research in high risk populations to a focus on reduction of malaria transmission. This will involve applied health research that is closely linked to programme implementation.

**d) Tuberculosis Control** (Cuevas, Squire, Theobald et al)

Tuberculosis is still one of the main causes of adult death in the world. The tools for prevention, diagnosis and treatment are still inadequate and health systems face enormous bottlenecks that hinder its control. LSTM's TB research focuses on generating key evidence to improve the smear-diagnosis of patients attending microscopy centers in resource poor settings, which contributed to several WHO policy changes in recent years including a reduction in the recommended number and collection sequence for collection of sputum specimens.[17]

It is acknowledged that one third of the TB cases occurring each year are not reported by National Programmes and that limited access to diagnosis is a key contributor, often with catastrophic economic costs for the patient, the family and the economy. Modelling work developed by LSTM has highlighted that new diagnostics would have limited impact if these tools are not implemented within systems that increase their accessibility to the place of need.[18,19] We demonstrated that approaches that are closer to the community than stationary health services can dramatically increase access to diagnosis and treatment of disadvantage populations.[20,21] LSTM work focusing in close to community approaches for TB has been highlighted as a trail-finder by the World Bank and the Global Fund and was presented at the World Health Assembly as means of increasing service access for women.

**e) Adolescent Sexual Health** (Obasi et al)

Improving adolescent reproductive health (ARH) in LMIC is key to their development. Pregnancy related death is the single biggest cause of death amongst girls aged 15-19 [22] and rates of sexually transmitted infections (STI) are at their peak among adolescents and young adults. However, the cultural, social and biological drivers of reproductive ill health in this age group are complex. Applied research is critical to understanding and addressing this complexity, as demonstrated by the LSTM-led evaluation of the scale-up of the *Mema kwa Vijana* (MkV) multicomponent ARH intervention in Tanzania (MkV2).[23]

The MkV intervention [23] informed the development of Government of Tanzania Youth Friendly Services guidelines and was adopted by WHO as a best practice intervention. [24] The MkV2 programme evaluated a 10 fold scale-up of the MkV intervention through existing government health and education systems. In addition to evaluation of the process of implementation, this research uniquely examined the local government policy process related to scale up and informed the 2013 UNESCO consultation on sexuality education.[26-28]

The implementation research in MkV2 highlighted the need and potential strategies for better integration between intervention settings, which is the focus of research led by LSTM through the *Int*HEC EU-funded consortium and illustrates how Delivery feeds back to Applied Research (Fig 5 above). Outputs from this research range from the first description of the reproductive outcomes and practices of very young adolescent girls (aged 11-15) in Niger, to an evaluation of mobile phone technology as a means of improving the integration of close to community providers in reproductive health service provision in Tanzania.

**f) HIV Testing & Counselling** (Taegtmeyer et al)

There is increasing evidence for the importance of anti-retroviral therapy in both improving the health of HIV-infected individuals and preventing new infections.[29] With large ‘testing gaps’ remaining in countries with high HIV prevalence, policy makers urgently need evidence on the models and approaches to the scale-up of HIV Testing & Counselling (HTC).[30] LSTM has spearheaded HTC scale-up through combining applied health research with policy influence and partnership with governments and NGOs that deliver these services. It has demonstrated effective quality assurance systems for HTC scale up in Kenya, co-authored a number of WHO documents on quality and developed an international practical manual on home-based HTC.[31] In April 2013 LSTM organized and co-hosted the First International Symposium on the legal, ethical, gender, human rights and public health aspects of HIV self-testing [32] and is working on a range of studies to address research gaps in this area, including on target product profiles of diagnostics, on couples testing, on the potential for social harms and on the uptake of counselling.

All of the above areas of work remain priorities for LSTM and will be carried forward in synergy with the outcomes of the 2014 CAHRD Consultation.

## Emerging outcomes of the Consultation

Bearing in mind the overall **Aim** to plan pathways towards solutions for challenges on the 10 to 20 year horizon, the following are initial reflections on progress towards the **Objectives**. At this stage these are very much in draft form and outlined here to stimulate further discussion, refinement and change in the course of the work on 12^th^ & 13^th^ June.

**a) Presenting concrete options for the further growth and development of work within the selected Workstreams**.

**Table 1:** Summary of options for further growth and development of work within the four Workstreams

Lung Health	i. Work towards better understanding of the health risks associated with Household Air Pollution (HAP), and what level of reduction of HAP is necessary to improve health.
	
	ii. Better evidence on which interventions for reducing household air pollution (technologies [e.g. stoves], fuels, ventilation, behaviour) are most effective at reducing HAP.
	
	iii. Community burden of chronic respiratory diseases in relation to epidemiological and socio-economic factors
	
	iv. Developing and testing health system diagnostic and clinical management pathways for patients with chronic cough, including provision for acute episodes
	
	v. Studies and systematic reviews of patient costs associated with chronic non-communicable respiratory disease
	
	vi. Work towards deeper understanding and effective measurement of catastrophic care-seeking costs
**Maternal & Newborn Health (MNH)**	i. The validity of MNH as a ‘litmus test’ for Universal Health Coverage (UHC)
	
	ii. Design and implementation of a single essential minimum health care package for mothers and babies, including understanding whether such package should include (e.g.) more gynaecological care, cancer screening & mental health
	
	iii. Models for the uptake, establishment and maintenance of audits of maternal deaths and stillbirths to improve quality of care, including new classification systems for attribution of cause of death and factors associated with death
	
	iv. Better understanding of the term “operations research” and more use of operations research in design and implementation of care packages to improve MNH

**Neglected Tropical Diseases (NTD)**	i. A new suite of tools and approaches for: sensitive surveillance of NTD transmission, decision support technology to facilitate effective responses to surveillance data, reduction of transmission (both vector control and chemotherapy of infected individuals)
	
	ii. New, better integrated health systems and transmission models for local decision making in policy and practice, along with capacity to develop and use these models.
	
	iii. Developing packages of care for the management of disability and promotion of mental health for those who live with chronic disabling and disfiguring consequences of NTD.
	
	iv. Identifying and controlling disease in hard-to-reach foci, along with maintaining momentum

**Health Systems**	i. Identifying best approaches to motivate, retain, and support different types of female and male close to community (CTC) providers as a key part of progress towards UHC
	
	ii. Working on opportunities for CTC providers to better address gendered social determinants of health at community level, and to promote effective multi-sectoral engagement
	
	iii. Developing human resource management systems, promoting health workforce supply, and improving health workforce performance in fragile and conflict-affected states (FCAS)
	
	iv. Wider use of a systematic, evidence-based approach for designing health research capacity strengthening (RCS) programmes, including implementation of common frameworks and use of monitoring indicators to capture impact on health systems and outcomes

b) Identifying cross-cutting themes and areas for collaboration across and between the Workstreams

At this stage, the following cross-cutting themes are emerging from the discussion papers and present opportunities for closer collaboration and sharing of experience and expertise between the four Workstreams:

i. **Equity impact** (including poverty status, gender, and intra-household dynamics) on access to healthcare, and the pivotal roles of focusing on human resources for health (in the broadest sense: CTC, capacity strengthening, & approaches to human resource management) in promoting equity in access. The importance of improving the measurement and understanding of costs to patients of healthcare utilization in relation to income.

ii. Devising sustainable approaches to **health service provision for chronic ill-health and disability** will be a major challenge in the 10-20 year horizon. While this need is increasingly articulated for the non-communicable diseases, there is a less recognised need emerging for the management of the chronic effects of all diseases in terms of disability and morbidity such as those associated with the NTD's.

iii. the need for **multi-sector interventions** to tackle the broad determinants of health leads to the observation that there are very few models of effective multi-sector engagement.

iv. The need for new and intensive **multi-disciplinary approaches** in applied health research and delivery, including a focus on capacity strengthening as a critical bottleneck between research and improved health.

v. Delivering sustainable, affordable, healthcare and **disease prevention programmes** really **close to communities**.

vi. Developing health systems in **conflict affected and fragile states** and marginalized populations.

vii. The need for **new tools**, applied research methods, locally tailored **modelling** approaches, technologies and strategies in the areas of diagnostics, treatment, health system functioning, and information systems.

**Figure 8:**
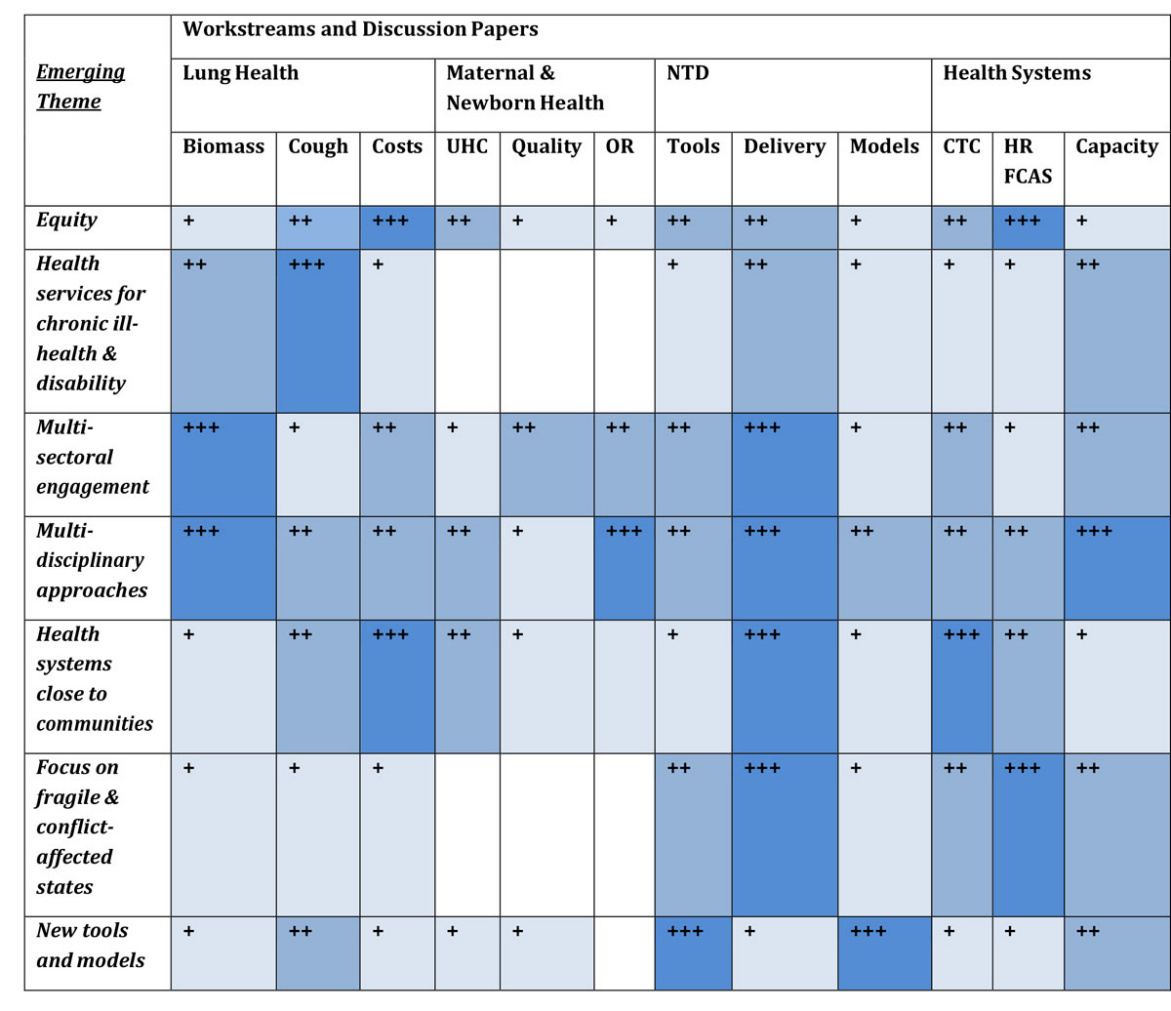
Cross-cutting themes in relation to Workstreams and Discussion Papers

These emerging themes resonate to differing extents with the Workstreams and Discussion papers as illustrated in Figure 8.

c) Identifying areas for collaboration between the 2014 Workstreams and existing Applied Health Research & Delivery Activities

**Figure 9:**
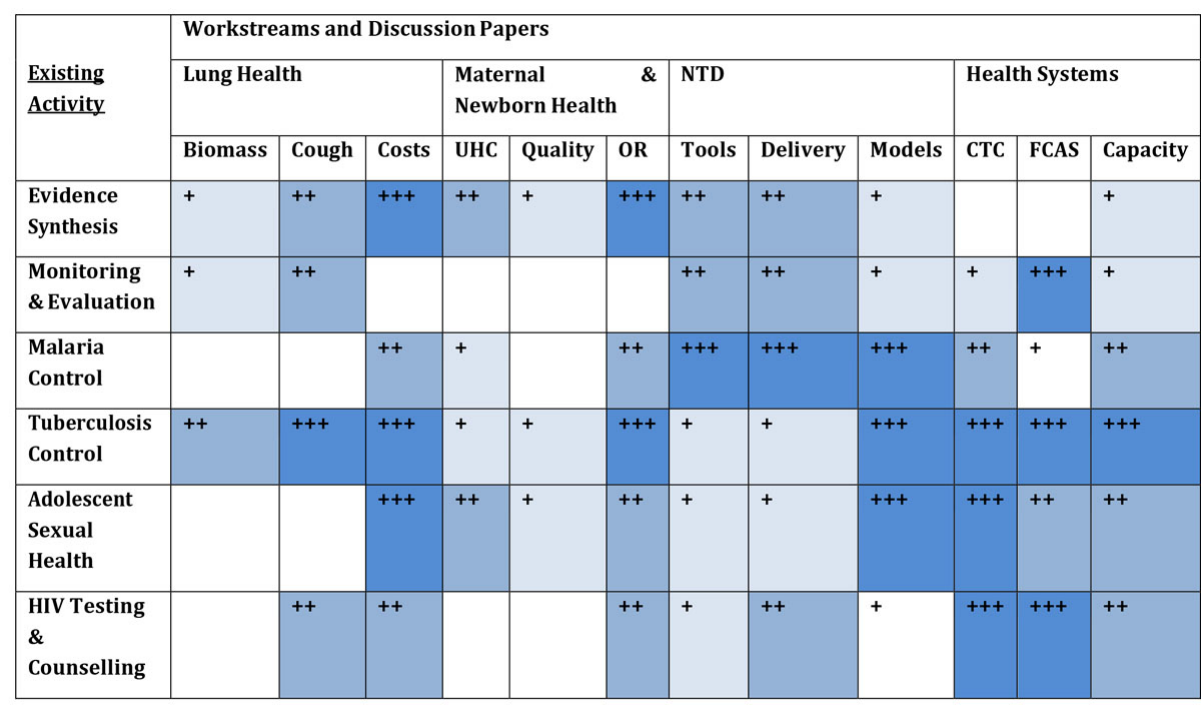
Intersections between Workstreams and existing Applied Health Research and Delivery Activities

Initial intersections between existing Applied Health Research & Delivery topics and the four Workstreams have been mapped in Figure 9.

d) Strengthening existing collaborations and forging new collaborations with global partners

Awaits results of further discussion on 12^th^ & 13^th^ June

## Taking forward the strategic direction

A number of processes are suggested to help take forward the ideas and plans from the Consultation:

a) **Evidence Synthesis**. Some questions emerging from the discussion papers could benefit from more formal systematic reviews. For example the work on catastrophic costs calls for a systematic review of studies of patients costs incurred during care-seeking for chronic respiratory diseases.

b) **Focussed Discussion Workshops**. Some of the options are likely to need further work after the Consultation. Individuals working in the Consultation Workstreams and individuals working on existing activities and those interesting in exploring more work on the cross-cutting themes could consider this option.

c) **Grant writing panels**. Some areas of activity could be matched to existing funding schemes and taken forward in the short term.

d) **Publications**. Some of the discussion papers could be further developed for publication. Examples include the work of the BREATHE Consortium in putting together a series for the Lancet Respiratory Medicine.

## Competing interests

There are no competing interests

## Declarations

This article has been published as part of *BMC Proceedings* Volume 9 Supplement 10, 2015: The 2014 CAHRD (Collaboration for Applied Health Research & Delivery) Consultation. The full contents of the supplement are available online at http://www.biomedcentral.com/bmcproc/supplements/9/S10.
